# Integrating Precision Livestock Farming and Genomic Tools for Heat Stress Mitigation in South African Dairy Cattle

**DOI:** 10.3390/ani16060947

**Published:** 2026-03-18

**Authors:** Mokgaetji Lebogang Papo, Keabetswe Tebogo Ncube, Simon Lashmar, Mamokoma Catherine Modiba, Bohani Mtileni

**Affiliations:** 1 Department of Animal Science, Tshwane University of Technology, Pretoria 0007, South Africa; mokgaetjilebogang@gmail.com (M.L.P.);; 2 Research Centre for Plant Metabolomics, Faculty of Science, University of Johannesburg, Auckland Park, P.O. Box 524, Johannesburg 2006, South Africa; 3 Agricultural Research Council, Animal Production, Private Bag X2, Irene, Pretoria 0062, South Africa; lashmars@arc.agric.za

**Keywords:** cattle well-being, climate resilience, genomic analysis, heat stress, precision livestock farming, selection signatures, sensor technologies

## Abstract

Heat stress is a significant problem in dairy production that has detrimental effects on milk production, animal well-being and reproductive function. These effects are predicted to worsen due to climate change. With a focus on South African production systems, this review assesses the potential of combining precision livestock farming (PLF) and genomic selection (GS) technology to identify, measure and reduce heat stress in dairy cattle. In addition to PLF tools like wearable sensors, rumen boluses, infrared thermography, GPS- and weather-based decision-support systems, pertinent literature was reviewed to evaluate genomic approaches such as heritability estimates and genome-wide association studies identifying selection signatures for thermotolerance. While advances in genomic techniques have improved the identification of thermotolerance markers and the accuracy of breeding values for heat tolerance, evidence from recent studies shows that PLF technologies can accurately detect early physiological and behavioural indicators of heat stress in real time. The ability to select climate-resilient animals under realistic farm conditions is improved by combining high-resolution phenotypic data from PLF systems with genetic data. Overall, the review concludes that combining PLF and GS provides a useful and complementary approach to enhance the detection of heat stress, facilitate well-informed management choices and hasten the development of thermotolerant dairy cattle, all of which contribute to more sustainable dairy production under rising temperatures.

## Simple Summary

Heat stress is a significant problem in dairy farming, which lowers milk output, negatively affects health and reproduction and makes cows less comfortable. As climate change results in more frequent and severe heat episodes, these issues are predicted to worsen. To effectively identify, control and lessen heat stress in dairy cattle, this review looks at how contemporary breeding techniques and digital animal monitoring technology can be used. According to research, devices such as temperature-monitoring cameras, animal-mounted sensors and automated weather-based systems can identify early changes in body temperature and behaviour related to heat stress. Furthermore, cattle that are inherently more tolerant of high temperatures can be identified, and farmers may make better management decisions, enhance animal welfare and sustain productivity in hot weather by combining enhanced breeding techniques with ongoing animal monitoring. This strategy promotes more sustainable dairy production, enhanced food security and increased livestock systems’ resistance to climate change.

## 1. Introduction

Heat stress in dairy cattle is a growing global concern, particularly in the context of climate change, where increasing ambient temperatures and the frequency of extreme weather events compromise animal health, milk production and overall farm profitability. Over the past 50 years, South Africa’s mean annual temperature is thought to have risen by at least 1.5 times the world’s average, or 0.65 °C [1]. Heat stress has been associated with reductions in milk yield in Holsteins under severe conditions in South African production systems, and summer fertility rates can decline by as much as 40% due to elevated thermal loads [2]. Moreover, provinces such as Limpopo, Mpumalanga and Northwest routinely experience temperature humidity index (THI) values exceeding 72 during summer, placing animals in chronic thermal discomfort [3,4]. It is anticipated that the frequency and severity of extreme weather occurrences will rise in the twenty-first century. If the region’s vulnerability is not significantly reduced, it will become more vulnerable to extreme weather events, particularly temperature extremes. Dairy industries worldwide are therefore under increasing pressure to adopt climate-resilient production strategies that integrate both technological and genomic innovations. Several precision livestock farming (PLF) technologies designed to maximise agricultural processes are designed to identify animal behavioural and physical alterations consistently and instantly [5]. PLF explains how sensor technologies, associated algorithms, interfaces, and applications are used in animal husbandry. All animal production systems employ it, but dairy farming is where it is most widely discussed. PLF is evolving swiftly and is transitioning from health alerts to an integrated system of decision-making. In addition to external data, it contains data from animal sensors and production. Only a portion of the numerous commercial applications that have been suggested or made accessible have undergone scientific evaluation; as a result, the true effects on animal welfare, production and health are still mostly unknown. While some technologies, such as sensors and computer vision for calving and oestrus detection, have been widely used, other methods are being adopted more slowly to reduce heat stress in dairy cattle, thereby making dairy farming more effective [6]. PLF provides prospects for the dairy industry through early disease diagnosis, more objective and consistent data collection on animals, risk prediction for animal health and wellbeing, improving animal production efficiency, and objectively determining animal emotional states.

Genomic selection (GS) predicts an animal’s breeding value using rich genome-wide DNA marker data without waiting for progeny testing. GS produces significantly faster genetic gain than traditional pedigree-based selection by shortening the generation interval and improving selection accuracy by training prediction models on animals with both genotypes and documented traits [7]. Heat stress decreases feed intake, milk yield and fertility and raises health issues, making it a significant climate-sensitive limitation for dairy production. Because heat tolerance exhibits genotype × environment (G × E) interactions and is partially heritable, genomic selection can help build herd resilience to rising temperatures by identifying and selecting animals that maintain productivity under heat load [8]. This is especially true when combined with large phenotypic datasets, environmental descriptors (such as temperature–humidity index), and G × E models. Cows projected to be heat-tolerant by genomic breeding values lose less milk and experience smaller increases in core temperature during heat events, according to experimental and large-scale studies. There are unique advantages and disadvantages to several heat stress mitigation techniques for dairy cattle, especially in South Africa. Although genetic development is slow and may include unfavourable correlations or trade-offs with economically significant production qualities [9,10], genetic selection for thermotolerance provides a cumulative and permanent solution that improves long-term resilience [11]. While real-time monitoring of physiological and behavioural markers is rendered accessible by precision livestock farming (PLF) technology, their efficacy is dependent on dependable infrastructure, data integration, technical knowledge, and financial investment [12]. Although dietary changes and housing adaptations can offer rapid and practical alleviation from heat stress, their effects are frequently context-specific, can raise operating expenses and do not result in long-term adaptive change [12]. These strategies can work in conjunction to improve overall system resilience by combining short-term environmental and management changes with long-term genetic improvement. However, widespread adoption of integrated solutions may be limited, especially in production systems with limited resources, due to the need for coordinated planning, capital investment, and technical capacity. This review seeks to evaluate how the combination of genomics and PLF technologies can be applied to detect, quantify, and mitigate heat stress in dairy cattle, thereby improving animal welfare and productivity.

## 2. Methodology of Literature Search and Selection

A systematic literature search approach was implemented to perform this narrative review to ensure comprehensive coverage of peer-reviewed research on mitigating heat stress in dairy calves. For publications published between 2000 and 2024, electronic databases such as Scopus, Web of Science, PubMed, and Google Scholar were searched. Heat stress, “dairy cattle,” “thermal neutral zone,” “temperature–humidity index,” “precision livestock farming,” “infrared thermography,” “wearable sensors,” “genomic selection,” “GWAS,” “heat tolerance,” and “South Africa” were among the keywords that were used in different combinations. Studies were incorporated if they (1) had an emphasis on dairy cattle, (2) reported responses to heat stress that have been reported on a physiological, behavioural, genetic, or management level, (3) evaluated genomic tools or precision livestock farming technologies and were published in English-language peer-reviewed publications. Studies that investigated non-dairy species, non-peer-reviewed papers, and grey literature were not included. To increase contextual relevance to South African production systems, studies relevant to subtropical and semi-arid regions were prioritised.

## 3. South African Dairy Industry

South Africa contains several dairy cattle breeds that perform similar functions in various ways. It is commonly believed that the Dutch East India Company introduced dairy cattle to South Africa in the 17th century to supply fresh milk to ships passing the Cape of Good Hope. Research indicates that the first Holstein cattle were introduced in 1850, whereas the years 1881 and 1890 have been reported as the dates for the arrival of the first Jersey and Ayrshire cattle in South Africa, respectively [13]. Today, these three dairy breeds predominate in South Africa’s dairy farming systems and contribute to diverse production goals, each offering distinct advantages. Holsteins have high-volume milk production; Jerseys have higher butterfat milk, and Ayrshires have better milk quality.

Holstein cattle are by far the most popular dairy breed worldwide due to their remarkable milk production. Due to decades of selective breeding for volume, some claim that Holsteins may produce an average of 10,000 L (or more) per lactation. Despite having a lower butterfat concentration (usually between 3.5 and 4%), their milk is the foundation of large-scale commercial dairy operations due to its sheer volume [14]. According to recent reports, their milk quality has even improved, with increases in the percentages of fat and protein and decreases in the somatic cell count (a health indicator) [15]. However, there are trade-offs associated with their high milk production: they require a high-quality diet and attentive care, and their leg and udder conformation may have an impact on their longevity.

Jersey cows, on the other hand, are significantly smaller yet yield milk that is remarkably high in content, particularly protein and butterfat. Their milk is particularly valuable for high-fat dairy products like cheese, butter and cream because their butterfat concentration can reach 5–6%. Jerseys are a recommended breed for farms that prioritise milk quality over mere volume since they are more feed-efficient (per unit of milk solids) and adapt well to pasture systems [14].

Ayrshire cattle provide a compromise, producing moderate amounts of milk with protein and fat concentrations halfway between those of Jerseys and Holsteins [16]. Because the fat particles are evenly distributed, their milk has a nice, “silky” quality. Ayrshires are very sturdy and resilient; their sound udders, strong legs and grazing efficiency make them suitable for a range of climates and pasture-based agricultural systems [17]. Their longevity and low somatic cell count, which is a gauge of udder health, help farmers reduce costs on replacement [18].

Holsteins dominate high-volume production systems and provide the vast majority of the fluid milk [19]. Due to their rich milk composition, Jerseys make a disproportionate contribution to the manufacture of speciality dairy products [16,20], whereas Ayrshires’ adaptability and health resilience support more flexible and sustainable production systems. The dairy industry can maximise production across various scales, climates, and market needs because of this diversity. Additionally, breeds are frequently cross-bred or selected [21,22] in breeding projects to balance productivity and sustainability because each breed has unique genetic attributes, such as yield, fat content and disease resistance. In this regard, the effects of heat stress on dairy cattle appear as interconnected physiological, morphological and behavioural responses [23,24], all of which help regulate body temperature and reduce thermal load.

### 3.1. Heat Stress in South African Dairy Production Systems

South Africa has varying agro-climatic zones, with the Western Cape experiencing Mediterranean climates while KwaZulu-Natal, Limpopo and North-West provinces experience subtropical and semi-arid conditions [25]. These regions influence the severity and length of exposure to heat stress. In the Western Cape, where seasonal heat waves are becoming more frequent [26,27], pasture-based systems predominate. On the contrary, hotter inland areas, where high ambient temperatures and humidity combine to raise the Temperature–Humidity Index (THI) above crucial thresholds for prolonged periods of time [28,29], are more likely to use feedlot-style systems and total mixed ration feeding strategies (TMR) [30].

Vulnerability is additionally influenced by breed distribution. Due in part to variations in metabolic heat output and surface-area-to-mass ratios, Holstein cattle—which are frequently used for their high milk production—are more vulnerable to heat stress than Jersey cattle and crossbred populations [31]. South Africa’s adoption of technology remains uneven. While large commercial farms have begun utilising automated milking systems and sensor-based monitoring technologies [32,33], small- to medium-sized producers frequently encounter limitations of infrastructure, electricity reliability, data administration capacity, and capital expenditure, regardless of whether large commercial farms have started utilising automated milking systems and sensor technology [34]. These contextual realities necessitate scalable, cost-sensitive solutions tailored to local climatic and socio-economic conditions.

### 3.2. Heat Stress in Dairy Cattle

Heat stress is a condition that develops when an animal’s body cannot sustain its normal temperature because of high external heat. It occurs in dairy cattle when they absorb more heat from their surroundings and internal bodily functions than they can expel through respiration, perspiration or other cooling activities. High external heat can arise from direct solar radiation, high ambient temperatures, elevated humidity, warm winds, or heat emitted from housing and equipment [35]. Cows that are under heat stress attempt to stay cool by increasing their respiration rate, consuming less feed and altering their behaviour (e.g., standing more and eating less) [12]. These changes frequently result in poorer milk production, decreased fertility, a compromised immune system, and generally lower output. The Temperature–Humidity Index (THI), a metric that estimates an animal’s degree of thermal discomfort by combining air temperature and humidity, is frequently used to quantify heat stress. For dairy cattle, a THI around 72 is typically regarded as stressful [36], though the cut-off may differ depending on the breed and level of acclimatisation. Numerous environmental elements, including care, nutrition and seasonality, have an impact on farm animals, and these impacts have a complex structure. A dairy cow’s thermoneutral zone, or the range of air where the animal produces the least amount of heat and has the most energy available for milk production, ranges between 5 and 25 °C and animal welfare may suffer if this range is exceeded. After all, the animal may not be able to disperse heat that is generated or absorbed metabolically, and thermal balance may not be maintained [37]. All of this includes the physiological, morphological and behavioural reactions that animals have while they are under heat stress.

Breed-specific differences in milk production traits, frame size, and physiological adaptability are central to understanding heat tolerance in dairy cattle [12,32]. Holstein cattle, despite their globally recognised high milk yield, exhibit low resilience to thermal stress due to their large body mass, high metabolic heat production and limited efficiency of heat dissipation relative to their heat load [32,38]. Although evaporative cooling through respiration and sweating contributes to thermoregulation, it represents only a portion of total heat loss, with radiative and convective pathways accounting for a substantial proportion under moderate conditions [38,39]. However, as ambient temperature and humidity increase, the effectiveness of radiative and convective heat loss declines, placing greater reliance on evaporative mechanisms [39]. In high-producing Holsteins, the imbalance between metabolic heat production and total heat dissipation capacity often results in decreased fertility and productivity in hot climates [34,40]. Jersey cattle, in contrast, produce lower volumes of milk but with higher fat and protein content, while their smaller body size, thinner skin, and short coat confer superior heat dissipation and adaptability to a tropical environment [41]. Ayrshires represent an intermediate profile, with strong milk performance and moderate thermotolerance, reflecting their origins in temperate regions but some adaptability in warmer systems.

Early research suggests that the Jersey breed may be more heat-tolerant than the Holstein breed for milk yield [42], even though the relationship between the breed and response to heat stress has not been robustly established in modern dairy cattle [42]. Therefore, heat stress is of major concern and must be considered when planning production systems and farm management. While Holsteins and Jerseys remain the dominant purebred dairy cattle, there is a growing trend among producers towards using crossbreeds to better address market and environmental demands. Research indicates that on hot days [42], Jersey cows’ heat stress indicators show lower levels, indicating a stronger capacity to withstand heat. Their smaller body size may be a primary contributing factor, as it confers a higher surface area to body weight ratio, aiding heat dissipation. The heat burden in Holstein cows is further compounded by higher milk output and the associated metabolic load. Additional physiological and morphological factors influencing breed resilience include skin thickness (thinner in Jerseys), subcutaneous fat deposition (lower in Jerseys) and hair coat characteristics (lighter colour and shorter length in Jerseys) [43] as shown in Table 1. It is generally accepted that animals with smaller fluctuations in core body temperature under heat load are better suited to hot environments. These physiological advantages suggest that Jerseys may be more suitable for the hotter agro-ecological zones of South Africa.

#### 3.2.1. Physiological Responses

Dairy cattle under heat stress experience physiological imbalances that impact their body temperature, heart rate, respiration, rumination time and reproductive efficiency. Cattle have trouble maintaining thermal homeostasis when outside temperatures rise beyond 30 °C or the Temperature Humidity Index (THI) rises above 80. As a result, their body and skin temperatures rise rapidly, they breathe rapidly and pant to release heat [37,44]. Compared to more heat-tolerant breeds such as Jerseys or indigenous cattle, high-producing breeds such as Holstein cattle are especially susceptible to these physiological reactions as their increased metabolic heat output exacerbates increases in body temperature and respiration rate [44]. Through a shift in blood pH equilibrium, this hyperventilation may result in rumen acidosis. While rumination time, which is a crucial digestive and milk production activity, decreases as THI rises, indicating decreased feed degradability and productivity, cows exposed to high temperatures also exhibit elevated heart rates, especially when not shaded. Prolonged exposure to heat stress damages oocytes and embryos, lowers conception rates and disrupts hormonal balance as plasma progesterone levels drop, impeding follicular development before ovulation, all of which hinder fertility and reproduction [45]. These combined physiological implications underline the significant impact of heat stress on dairy cattle health, production and reproductive efficiency, especially in hot and humid settings.

#### 3.2.2. Morphological Responses

Due to physical characteristics including coat colour, thickness and hair density, various breeds of cattle react differently [37] to heat stress. Breeds with lighter coats, like white or red, can be more heat-tolerant because they reflect more sun radiation than dark-coated animals, which absorb more heat. The cooling benefit of lighter pigmentation is demonstrated by studies that show black cattle’s body temperatures can rise by 4.8 °C in direct sunshine [37], but white cattle’s body temperatures only rise by 0.7 °C. By enhancing heat dissipation, coat traits such as short, thin hair and fewer follicles increase tolerance to hot temperatures [46]. Holstein cattle with certain coat colours and physical characteristics are more likely to survive and tolerate heat in tropical climates [46], highlighting the importance of coat characteristics and pigmentation in thermoregulation and environmental adaptability.

**Table 1:** Comparative characteristics of selected dairy breeds in relation to heat tolerance.

Breed	Milk Yield	Milk Fat%	Frame Size	Heat Tolerance	Skin/Fur Traits	References
Holstein	High (6000–7000 kg per lactation)	3.6	Large	Lower	Thick skin, long fur	[38,43]
Jersey	Lower (4500–5000 kg per lactation)	4.8–5.2	Small	Higher	Thin skin, short fur	[29,31,32]
Ayrshire	Lower than Holsteins	4.2	Medium–Large	Moderate	Thick skin, short fur	[47,48]
Guernsey	6000 kg per lactation	4.5–5	Medium	Moderate	Think skin, short sleek fur	[48,49]

#### 3.2.3. Behavioural Responses

To manage heat stress, dairy cattle display a variety of behavioural modifications such as adjustments to their standing, lying, drinking and feeding habits that are intended to lower their body heat burden and preserve comfort. Cows stand more and lie less in hot weather with elevated temperature humidity index (THI) readings [50], which helps them release body heat but decreases their rest period and milk output. Standing and lying are important markers of thermal stress and well-being since research indicates that when THI rises, lying time may decrease by up to three hours per day [51]. Due to the requirement for evaporative cooling, cows’ water intake increases dramatically with THI. They also drink more often, spend longer time at the drinkers and exhibit more competition, particularly in the absence of shade. Conversely, when cows experience heat stress, they limit their dry matter intake (DMI) to lessen the amount of heat they produce metabolically [51]. This reduced appetite and rumination results in decreased saliva buffering in the rumen, having an adverse effect on digestion, milk production and energy balance [52]. Overall, these behavioural responses show how dairy cattle actively modify their daily life activities to deal with heat stress at the cost of their health and production.

## 4. Precision Livestock Farming

Before the development of precision livestock farming technologies, traditional methods such as ventilation, shading, and watering techniques were and are still used to reduce heat stress and preserve animal welfare in hot environments. While these strategies remain important, Precision Livestock Farming (PLF) refers to the application of advanced sensor technologies, data analytics, and automation systems to monitor and manage individual animals in real time. By continuously capturing physiological, behavioural, and environmental data, PLF enables early detection of stressors, including heat stress, and facilitates timely and targeted interventions. In the context of climate change, PLF offers a promising strategy to enhance animal welfare, production efficiency, and farm sustainability in both intensive and extensive dairy systems. Its primary goal is to provide real-time alerts in cases of abnormalities, enabling prompt intervention while ensuring sustainability and animal welfare [53].

An example of PLF technology is respiratory rate monitors that automatically detect breathing frequency using micro-computers and thin-film pressure sensors, providing indicators of temperature and health status. Studies have demonstrated variations in respiration rate between shaded and unshaded calves, underscoring sensitivity to environmental stress [54]. Accelerometers use electromechanical sensors to record three-dimensional bodily motions, allowing identification of behaviours such as eating, drinking, resting, and ruminating. When combined with gyroscopes and magnetometers, these wearable collar technologies improve behavioural classification and enable early identification of stress, illness, or lameness [55].

Machine learning further enhances real-time herd-level analysis. The GPS collars and GIS systems track movement patterns, grazing behaviour, and activity, providing valuable welfare and energy-use information [56]. When paired with accelerometers, grazing zone detection accuracy can reach up to 95% [57]. These systems are complemented by infrared thermography (IRT), which remotely measures body surface temperature and correlates strongly with heat stress indicators such as THI [58]. Used together, these tools allow continuous, non-invasive monitoring of cattle physiology and behaviour, supporting timely heat stress mitigation and improved welfare [59]. As sensor precision and affordability improve, PLF is becoming increasingly accessible to South African dairy producers seeking climate-adaptive solutions. Genetic approaches complement these technologies by providing long-term strategies for selecting heat-resilient traits. Together, they form a proactive framework for mitigating heat stress and supporting climate-smart dairy farming.

A frequently used non-invasive method of detecting thermal stress is infrared thermography (IRT), which examines the distribution of surface temperatures. The orbital area is regarded as the most dependable zone of interest among anatomical regions due to its strong vascularization and low hair coverage, which improves the precision and reproducibility of temperature readings.

About 40–60% of cattle’s total heat dissipation occurs by radiative heat loss under thermoneutral settings. However, the relative importance of evaporative cooling processes increases with rising ambient temperatures. As a result, it is critical to consider environmental factors such as humidity and airflow while interpreting IRT data.

The Thermal Neutral Zone (TNZ) varies by breed, acclimatisation status, metabolic heat generation, and humidity conditions, and it therefore should not be regarded as a set range. Holstein cattle, for instance, could react to stress at lower THI thresholds than Jersey cattle, which are acclimated to warmer regions. To ensure data dependability, IRT device calibration, emissivity parameters (typically 0.98 for cow skin), and imaging distance standardisation are crucial.

**Figure 1:**
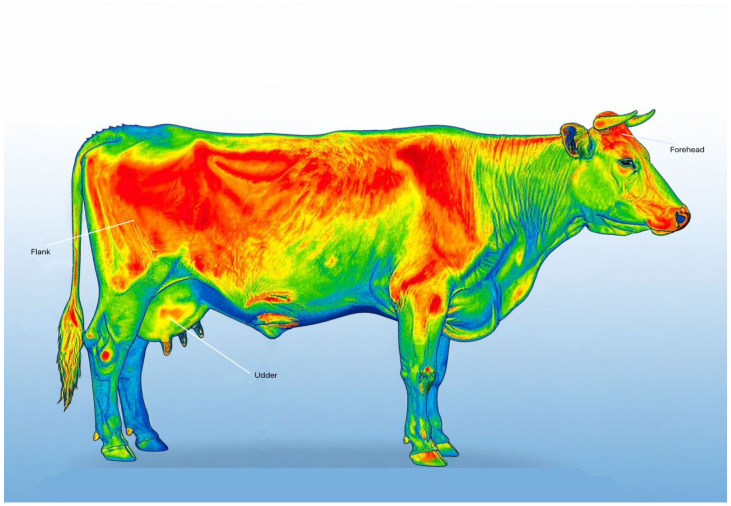
Key anatomical zones on dairy cattle are commonly assessed using infrared thermography (IRT), including the forehead, flank and udder. Surface temperature variations are visualised through colour gradients and are correlated with temperature–humidity index (THI) values to evaluate heat stress responses [AI-generated image].

Figure 1 is an AI-generated image which shows how infrared thermography can be used to detect key anatomical zones in cows for heat stress detection.

As the precision and affordability of sensor technologies continue to improve, PLF is becoming increasingly accessible to South African dairy producers seeking climate-adaptive solutions [60]. Complementing these technological advancements, genetic approaches offer long-term strategies for improving thermotolerance in dairy cattle through the identification and selection of heat-resilient traits. Together, these tools provide a proactive framework for mitigating heat stress, reducing economic losses, and supporting climate-smart dairy farming.

### 4.1. Precision Livestock Technologies to Mitigate Heat Stress

PLF is one of the most transformative innovations in the livestock industry. Advances in information and communication technologies, wireless networks, and internet access over the past two decades have enabled integrated application of multiple tools within unified systems [60,61]. Sensor-based strategies shift heat stress management to the individual animal level. Advanced monitoring permits targeted mitigation under heat stress conditions and supports the selection of climate-resilient cattle. Some automated techniques remain under evaluation, while others are validated for monitoring behaviour and health [62].

**Figure 2:**
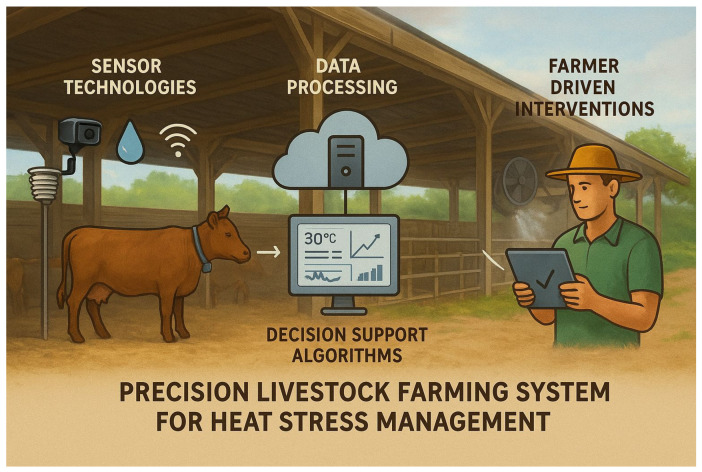
A schematic representation of a Precision Livestock Farming (PLF) system for heat stress management, illustrating the integration of sensor technologies, data processing (cloud/edge computing), decision support algorithms, and farmer-driven interventions [64].

The full potential of these technologies is realised when integrated into comprehensive management systems. Figure 2 illustrates a PLF framework linking sensors, data processing platforms, decision-support tools, and farmer interventions. Sensors continuously collect real-time data on behaviour, physiology, and environmental conditions [63]. Environmental monitoring includes temperature, humidity, air quality, and light intensity in barns and pastures. Devices may include wearable sensors, fixed monitoring systems, or aerial drones. Data are processed using artificial intelligence and machine learning to detect patterns and anomalies [64]. Integrated decision-support systems then generate actionable insights to optimise herd management, productivity, and welfare. One example of such a framework is demonstrated in Figure 2, which shows a PLF system for managing heat stress that links sensor data, processing platforms, decision support tools, and farmer interventions.

### 4.2. Opportunities and Limitations of PLF in South Africa

In South Africa’s dairy industry, PLF presents opportunities to improve sustainability, resilience, and productivity amid increasingly frequent heat stress. Wearable sensors, rumination monitors, activity trackers, and automated milk recording systems enable early detection of health, fertility, and physiological stress, particularly in intensive systems [65,66].

PLF also supports data-driven heat stress mitigation (Table 2). Continuous monitoring of body temperature, feeding behaviour, and milk yield allows adjustments to cooling, nutrition, and management strategies. Studies from developed and developing regions report reduced production losses and veterinary costs when PLF is integrated into herd management systems [67,68,69]. These benefits are especially relevant as rising temperatures threaten dairy cow performance and longevity in South Africa. However, adoption remains uneven due to structural and economic constraints. High investment costs, reliance on imported equipment, exchange rate volatility, and limited access to affordable finance restrict uptake, particularly among smallholder and emerging producers [70,71,72]. Infrastructure challenges, including unreliable electricity and limited rural internet access, further limit implementation.

Environmental pressures such as drought, water scarcity, and pasture degradation reduce the effectiveness of water-dependent cooling systems and precision technologies in arid and semi-arid regions [69,73]. Institutional barriers and limited digital literacy also hinder adoption, as many farmers lack access to specialised training and extension services [9,74,75]. Overall, PLF has significant potential to enhance heat stress management in South Africa, but widespread adoption will require coordinated policy support, financing mechanisms, and capacity-building initiatives.

**Table 2:** Key precision livestock farming (PLF) and management technologies for mitigating heat stress in dairy cattle: advantages, limitations, cost, and applicability in South Africa.

Technology	Advantages	Limitations	Cost Level	Suitability in South Africa	References
**Infrared thermography**	Non-invasive, rapid detection	Sensitive to environmental interference	Moderate	Suitable for large farms	[58,59]
**Wearable sensors**	Continuous Monitoring	Data management complexity	High	Large commercial systems	[56,61]
**Genomic selection**	Long term resilience	Requires phenotypic data and genotyping cost	High initial cost	Breeding programmes	[76,77]
**THI monitoring**	Low cost	Less individual specificity	Low	All Farm sizes	[28,29]

## 5. Genetic Assessment of Heat Tolerance in Dairy Cattle

Given South Africa’s increasingly severe climatic conditions, the use of genomic technologies offers a potent means of improving dairy cattle’s heat resistance. Herd adaptability can be increased by breeding animals with favourable alleles linked to resilience, productivity and thermoregulation through genomic selection [76]. In South Africa, an important basis for genomic studies is provided by national milk records and genetic improvement programmes run by Milk SA with technical assistance from the Agricultural Research Council (ARC) and industry partners. These programmes produce pedigree and phenotypic data that can be combined with genomic data to calculate genomic estimated breeding values (GEBVs) for heat-tolerance-related traits, making it easier to select cows that continue to produce optimally when exposed to elevated temperatures.

However, there are still a few locally established reference populations, especially for thermotolerance traits, which limit the use of genomic selection for mitigating heat stress in South Africa. Currently, most genomic predictions are based on marker effects from global datasets, which might not accurately represent genotype–environment interactions in the climate of South Africa [77,78]. The accuracy of genomic selection for heat tolerance in South African dairy cattle could be significantly increased by strengthening locally relevant genomic reference populations through the integration of phenotypes derived from precision livestock farming, such as body temperature, activity, and milk yield responses during heat events.

Genomic selection for heat tolerance in South African dairy cattle faces several obstacles despite its potential. These include the high cost of genotyping, the unequal use of genomic technology in commercial and developing dairy systems and the scarcity of phenotypic records related to heat stress. Furthermore, a limited number of extremely productive breeds, especially Holsteins and Jerseys, dominate South Africa’s dairy sector, potentially reducing genetic diversity for adaptive qualities. To overcome these obstacles, industry organisations like Milk SA, research organisations like the ARC [79,80] and dairy producers must work together to define climate-resilient breeding goals specific to South Africa and to extend recording systems.

### 5.1. Heritability Traits

Genomic selection provides a viable and sustainable method of improving heat tolerance in dairy cattle by identifying cattle with favourable genetic profiles for thermoregulation and production under heat stress. Heat tolerance-related traits have been shown in numerous studies to have low to moderate heritability, suggesting significant potential for genetic improvement through selection. While production-based measures of heat tolerance, such as milk yield decline under elevated temperature–humidity index (THI) conditions, have heritability estimates between 0.05 and 0.20, physiological indicators such as rectal temperature, respiration rate and body surface temperature typically have estimates between 0.10 and 0.30. Additive genetic components for milk, fat, and protein outputs under heat stress were found in US Holsteins and Jerseys in a wide genomic evaluation using reaction-norm models, suggesting the existence of genetic diversity that can be selected for. However, non-genetic (permanent environmental) impacts explained more of the variation in some attributes under heat stress in jerseys, indicating that genetic selection for heat tolerance may be more difficult for some production traits in this breed [81].

GEBVs can be used to select superior animals that sustain performance in high-temperature conditions, as research indicates that there is significant genetic variation for heat tolerance features. Studies have revealed that incorporating heat stress-related phenotypes to genomic prediction models, such as respiration rate, rectal temperature and milk yield responses to rising THI, significantly improves prediction accuracy when compared to traditional pedigree-based methods [82,83,84]. Holstein cows exhibit higher increases in rectal temperature and larger shifts in rumen fermentation profiles under heat stress, while Jerseys show physiological and microbial patterns linked to relatively better heat adaptation. These breed-specific reactions highlight how crucial it is to take genetic architecture and breed composition into account when using genomic selection for heat tolerance [85].

In South Africa, where there are varied dairy systems, these breed variations are significant. High-producing commercial herds are dominated by Holsteins, although in hotter, more humid climates, Jerseys and crossbreeds are becoming increasingly popular. Breed-specific responses can be incorporated into genomic selection models to increase accuracy and guarantee that genetic merit for output across climates is not compromised by selection for heat tolerance [81].

### 5.2. Genome-Wide Association Studies for Heat Stress Mitigation

Genome-wide association studies (GWAS) have emerged as a crucial technique to understand the genetic foundation of dairy cattle’s resistance to heat stress. GWAS provides an evidence-based strategy for enhancing resilience by identifying certain genetic loci linked to heat-affected physiological characteristics/quantitative phenotypes such as respiration rate, milk yield under heat stress and rectal temperature [86] by scanning the genome for single-nucleotide polymorphisms (SNPs) statistically associated with heat-affected phenotypes [43]. The identification of quantitative trait loci (QTLs) and candidate genes by GWAS provides mechanistic targets for selection and functional follow-up by connecting physiological responses (e.g., thermoregulatory capacity to molecular pathways) to molecular pathways which typically implicate heat-shock proteins, cellular stress response genes and metabolic regulators [87].

In terms of methodology, contemporary GWAS for heat tolerance use several improvements: (1) regression or reaction-norm models that calculate genetic sensitivity to the environment (e.g., change in milk yield per unit THI); (2) single-step genomic approaches that integrate genotype, phenotype, and pedigree for small or heterogeneous populations and (3) weighted/single-step GWAS or integration with transcriptomics (RNA-sequencing) to rank biologically significant loci. These methods improve biological interpretability and power, particularly in situations with sparse phenotypes or diverse settings [55].

GWAS can be used to address issues for South African dairy systems involving multibreed/admixed analyses because many herds contain crossbreds; genotype × environment (G × E) modelling to account for diverse climates and management, and accurate, continuous phenotyping of heat-response traits (using PLF sensors like IRT, RR monitors and accelerometers) [88]. In addition to identifying candidate genes for inclusion in genomic selection indices or marker panels appropriate for local germplasm, functional validation, such as superimposing GWAS findings with differential expression (RNA sequencing) or methylation studies, strengthens causal inference.

### 5.3. Selection Signatures and Runs of Homozygosity for Heat Stress Mitigation

Runs of homozygosity (ROH) and selection signature studies have emerged as crucial genomic methods for identifying genetic modifications that improve dairy cattle’s thermotolerance. ROH are continuous stretches of homozygous genotypes in the genome that reflect shared ancestry and are commonly used to estimate genomic inbreeding and identify regions potentially under selection. Selection signatures are detectable patterns in the genome that indicate past or ongoing natural or artificial selection, often highlighting regions associated with advantageous traits such as heat tolerance or productivity. By identifying genomic areas that have seen positive selection due to extended exposure to heat stress, these methods uncover potential genes that can be included in breeding plans. To find loci linked to heat adaptation, selection signature techniques like FST, integrated haplotype score (iHS), and cross-population extended haplotype homozygosity (XP-EHH) analyse allele frequencies or haplotype structures between populations exposed to various thermal settings [89]. Genes linked to immunological modulation, oxidative stress response and heat shock proteins (HSP70, HSP90) are found in many of these areas. These genes are essential for preserving cellular integrity under high ambient temperatures [87].

By locating lengthy homozygous regions that signify past selection or inbreeding events, ROH analysis enhances existing methods. It is possible to identify regions where selection has favoured alleles that provide resilience to heat stress by overlapping ROH hotspots with genes relevant to thermotolerance [90]. For instance, ROH and selection signature analyses in African cow populations have identified genetic areas related to immunological tolerance, metabolic control and sweating efficiency, highlighting the adaptive potential of native breeds like Afrikaner and Nguni [90,91]. Breeding programmes that preserve adaptive variety and boost productivity can be designed for South African dairy systems by combining ROH and selection signature results with GWAS and genomic selection. A sustainable route to producing dairy calves that are resilient and prolific in the face of growing climate stress is provided by this all-encompassing genomic strategy.

### 5.4. Challenges of Using the Genomic Approaches

Although heat stress has a negative impact on dairy cattle’s welfare and productivity, efforts to enhance genetics are made more difficult by the low to moderate heritability of heat tolerance traits. The success of conventional breeding programmes is limited by the heritability estimates for traits such as respiration rate, rectal temperature and milk output under heat stress, which range from 0.1 to 0.3. Furthermore, the complicated polygenic character of these traits presents difficulties for GWAS that seek to discover single-nucleotide polymorphisms (SNPs) linked to heat tolerance [86]. Finding reliable genetic markers is made more difficult by environmental variables, genotype-by-environment interactions and the requirement for high-quality phenotypic data.

ROH and selection signature analyses are useful methods for locating genomic areas linked to dairy cattle’s ability to withstand heat. Nevertheless, these techniques have drawbacks that may compromise their precision and usefulness. False positives could result from selection signatures identifying areas affected by past selection that have nothing to do with heat tolerance [92]. Additionally, in smallholder or resource-constrained systems, ROH analysis may be limited by population size, marker density and genetic diversity. Large, high-quality datasets are necessary for integrating these methods with GWAS, and functional validation of candidate genes is still difficult. Studies have shown, for instance, that selection signatures can identify potential genes for heat tolerance; however, the interpretation of these findings necessitates a detailed analysis of environmental conditions and the population’s historical background.

## 6. Integrating Precision Livestock Farming with Genomic Approaches for Heat Stress Mitigation

PLF technologies provide a practical way to monitor individual dairy cattle under various environmental conditions, therefore offering high-throughput phenotypic data essential for managing heat stress. Wearable sensors and automated monitoring systems from these technologies can continuously record physiological indicators, including respiratory rate, rectal temperature and activity levels. This real-time monitoring makes it feasible to obtain accurate, long-term data that demonstrates how animals react to heat stress throughout the day and in various seasons. These detailed phenotypic profiles offer a strong foundation for selecting animals with superior thermotolerance, allowing for specific breeding strategies and well-informed management decisions [62]. For example, research has shown that sensor-derived data might enhance genetic evaluations by delivering precise and fast phenotypic information, increasing the accuracy of genomic selection for traits related to heat tolerance.

Precision Livestock Farming (PLF) technologies offer a practical means of monitoring individual dairy cattle under varying environmental conditions, providing high-throughput phenotypic data critical for heat stress management. Wearable sensors and automated monitoring systems can continuously record physiological indicators such as respiration rate, rectal temperature, and activity levels. This real-time monitoring allows for the collection of precise and longitudinal data, capturing how animals respond to heat stress throughout the day and across different seasons. Such detailed phenotypic profiles provide a strong foundation for identifying animals with superior thermotolerance, enabling informed management decisions and targeted breeding strategies [93].

**Figure 3:**
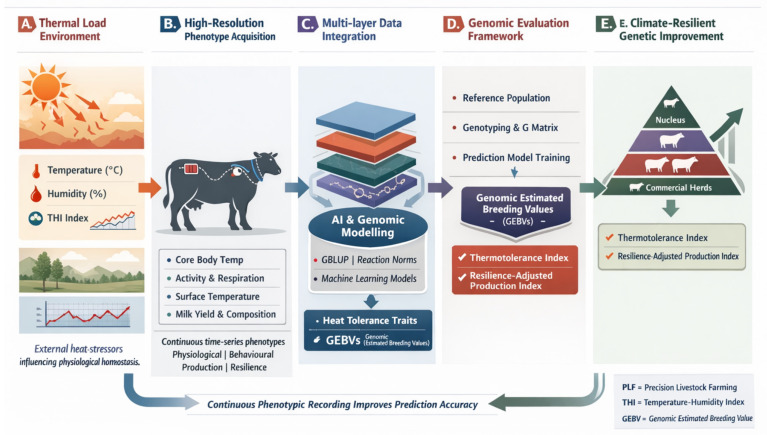
Conceptual integration of precision livestock phenotypes into genomic selection pipelines for heat stress mitigation [AI-generated image].

The integration of PLF data with genomic tools further enhances the identification of heat-tolerant animals (Figure 3). It is feasible to identify the genetic foundations of thermoregulation and identify genotype-by-environment interactions that affect responses to heat stress by connecting the sensor-derived phenotypes to genomic data. This approach strengthens genome-wide association studies (GWAS) and selection signature analyses, offering a more precise understanding of the genetic variation underlying heat tolerance. Because repeated assessments under various environmental conditions capture minute variations in animal performance that could otherwise go unnoticed, longitudinal data from PLF technology enhances the accuracy of these analyses [77].

Furthermore, combining PLF-derived phenotypes with genomic data improves the accuracy of genomic selection methods. If selection is focused solely upon productivity traits, traditional breeding techniques may not be able to identify animals that function well under heat stress. Breeding values that represent both thermotolerance and production performance can then be estimated by integrating high-resolution phenotypic data into genomic prediction models, and through this combination, breeders can maintain high milk yield and overall herd productivity while choosing sires and dams with superior genetic potential for heat resilience [33].

Ultimately, a sustainable and practical framework for reducing heat stress in dairy cattle was provided by the useful integration of PLF and genomic methods. While genetic selection guarantees sustained improvements in herd thermotolerance, real-time phenotypic monitoring facilitates rapid management actions. When combined, these technologies enable data-driven decision-making that promotes climate-resilient dairy farming systems, improves animal wellbeing, and sustains output in harsh climatic conditions [77].

## 7. Policy and Capacity Building for Adoption Involving the Role of Industry, the Dairy Industry and Government

The adoption of precision livestock farming (PLF) technologies in South Africa poses unique socioeconomic challenges due to the dual structure of the dairy industry, which consists of highly mechanised commercial farms and resource-constrained small- and medium-scale producers [94]. Existing gaps in productivity and competitiveness could be widened by unequal access to capital, technical knowledge, dependable electricity, and rural internet connectivity. In this regard, a major obstacle to the equal adoption of technology is the digital divide.

Data governance and ownership must also be carefully considered. Concerns about farm privacy, control over farm-generated data [71,95], and the commercial use of production data are raised by PLF systems’ growing reliance on cloud-based platforms and third-party analytics [96]. Large-scale adoption is rendered more difficult by infrastructure instability, especially interruptions in the electrical supply, which may call for low-cost and energy-efficient monitoring options tailored to local conditions.

From a breeding point of view, the maintenance of genetic variety within South Africa’s dairy population, which is primarily Holstein-based, must be balanced with selection for thermotolerance [71]. An excessive focus on single-trait selection may inadvertently threaten resilience, productivity, or fertility. To promote the responsible, inclusive, and sustainable adoption of PLF and genomic technologies in the face of changing climate conditions, supportive policy measures—such as targeted subsidies, extension services, and capacity-building initiatives—will be crucial.

## 8. Future Perspectives 

Combining genetic techniques and PLF technologies is a viable strategy for reducing heat stress in dairy cattle. To find reliable thermotolerance markers, future studies should concentrate on high-resolution, real-time phenotyping of physiological and behavioural responses in conjunction with genetic data like GWAS, selection signatures and ROH. By improving the utilisation of these datasets, machine learning and predictive analytics can facilitate the early identification of heat stress and well-informed management strategies. It is essential to include genotype-by-environment interactions and affordable, scalable PLF solutions, especially in systems with limited resources in South Africa and other African countries, where managing heat stress is hindered by a lack of feed and water.

## 9. Conclusions

Dairy productivity and welfare are still intensely threatened by heat stress, and conventional management techniques are not enough. By finding and spreading heat-tolerant characteristics while preserving productivity, sensor-based phenotyping combined with genomic selection can increase resilience. This comprehensive approach, which combines PLF technologies, genomic tools and sustainable management strategies, offers a way to develop high-performing, heat-resilient dairy herds in increasingly stressful climatic conditions, despite obstacles like the low heritability of thermotolerance traits and the requirement for large datasets.

## Data Availability

No data were used for the research described in the article.

## Abbreviations

The following abbreviations are used in this manuscript:

ARC	Agricultural Research Council
DMI	Dry Matter Intake
DNA	Deoxyribonucleic Acid
FAO	Food and Agriculture Organisation of the United Nations
FST	Fixation Index
GEBVs	Genomic Estimated Breeding Values
GIS	Geographic Information Systems
GPS	Global Positioning System
GS	Genomic Selection
GWAS	Genome-Wide Association Studies
G × E	Genotype by Environment Interaction
HSP	Heat Shock Protein
iHS	Integrated Haplotype Score
IRT	Infrared Thermography
PLF	Precision Livestock Farming
QTLs	Quantitative Trait Loci
RNA	Ribonucleic Acid
ROH	Runs of Homozygosity
SNPs	Single Nucleotide Polymorphisms
THI	Temperature–Humidity Index
TMR	Total Mixed Ration
XP-EHH	Cross-Population Extended Haplotype Homozygosity
